# Motor inhibition and its contribution to recovery of dexterous hand use after stroke

**DOI:** 10.1093/braincomms/fcac241

**Published:** 2022-09-23

**Authors:** Jeanette Plantin, Alison K Godbolt, Gaia V Pennati, Evaldas Laurencikas, Peter Fransson, Jean Claude Baron, Marc A Maier, Jörgen Borg, Påvel G Lindberg

**Affiliations:** Division of Rehabilitation Medicine, Department of Clinical Sciences, Karolinska Institutet, Danderyd University Hospital, Stockholm, Sweden; Division of Rehabilitation Medicine, Department of Clinical Sciences, Karolinska Institutet, Danderyd University Hospital, Stockholm, Sweden; Division of Rehabilitation Medicine, Department of Clinical Sciences, Karolinska Institutet, Danderyd University Hospital, Stockholm, Sweden; Division of Rehabilitation Medicine, Department of Clinical Sciences, Karolinska Institutet, Danderyd University Hospital, Stockholm, Sweden; Department of Clinical Neuroscience, Karolinska Institutet, Stockholm, Sweden; Department of Neurology, Groupe Hospitalo-Universitaire Paris Psychiatrie et Neurosciences, Université Paris Cité, Paris, France; Institut de Psychiatrie et Neurosciences de Paris, Inserm U1266, 102-104 rue de la Santé, Paris 75014, France; Université Paris Cité, CNRS, Integrative Neuroscience and Cognition Center, Paris, France; Division of Rehabilitation Medicine, Department of Clinical Sciences, Karolinska Institutet, Danderyd University Hospital, Stockholm, Sweden; Division of Rehabilitation Medicine, Department of Clinical Sciences, Karolinska Institutet, Danderyd University Hospital, Stockholm, Sweden; Institut de Psychiatrie et Neurosciences de Paris, Inserm U1266, 102-104 rue de la Santé, Paris 75014, France

**Keywords:** stroke, recovery, motor inhibition, hand, MRI

## Abstract

Recovery of dexterous hand use is critical for functional outcome after stroke. Grip force recordings can inform on maximal motor output and modulatory and inhibitory cerebral functions, but how these actually contribute to recovery of dexterous hand use is unclear. This cohort study used serially assessed measures of hand kinetics to test the hypothesis that behavioural measures of motor modulation and inhibition explain dexterity recovery beyond that explained by measures of motor output alone. We also investigated the structural and functional connectivity correlates of grip force control recovery.

Eighty-nine adults (median age = 54 years, 26% females) with first-ever ischaemic or haemorrhagic stroke and persistent arm and hand paresis were assessed longitudinally, at 3 weeks, and at 3 and 6 months after stroke. Kinetic measures included: maximal grip force, accuracy of precision and power grip force control, and ability to release force abruptly. Dexterous hand use was assessed clinically with the Box and Block Test and motor impairment with the upper extremity Fugl-Meyer Assessment. Structural and functional MRI was used to assess weighted corticospinal tract lesion load, voxel-based lesion symptom mapping and interhemispheric resting-state functional connectivity.

Fifty-three per cent of patients had severe initial motor impairment and a majority still had residual force control impairments at 6 months. Force release at 3 weeks explained 11% additional variance of Box and Block Test outcome at 6 months, above that explained by initial scores (67%). Other kinetic measures did not explain additional variance of recovery. The predictive value of force release remained significant when controlling for corticospinal tract lesion load and clinical measures. Corticospinal tract lesion load correlated with recovery in grip force control measures. Lesions involving the parietal operculum, insular cortex, putamen and fronto-striatal tracts were also related to poorer force modulation and release. Lesions to fronto-striatal tracts explained an additional 5% of variance in force release beyond the 43% explained by corticospinal injury alone. Interhemispheric functional connectivity did not relate to force control recovery.

We conclude that not only voluntary force generation but also force release (reflecting motor inhibition) are important for recovery of dexterous hand use after stroke. Although corticospinal injury is a main determinant of recovery, lesions to integrative somatosensory areas and fronto-parietal white matter (involved in motor inhibition) explain additional variance in post-stroke force release recovery. Our findings indicate that post-stroke upper limb motor impairment profiling, which is essential for targeted treatment, should consider both voluntary grasp generation and inhibition.

## Teaser

Plantin *et al*. report that grip force release, early after stroke, explains variance of dexterous hand use recovery not explained by conventional motor measures. Prolonged force release was related to corticospinal, somatosensory and fronto-striatal tract lesion. Thus, motor inhibition (subserving release) is essential for post-stroke recovery of dexterous hand use.

## Graphical Abstract

**Graphical abstract:**
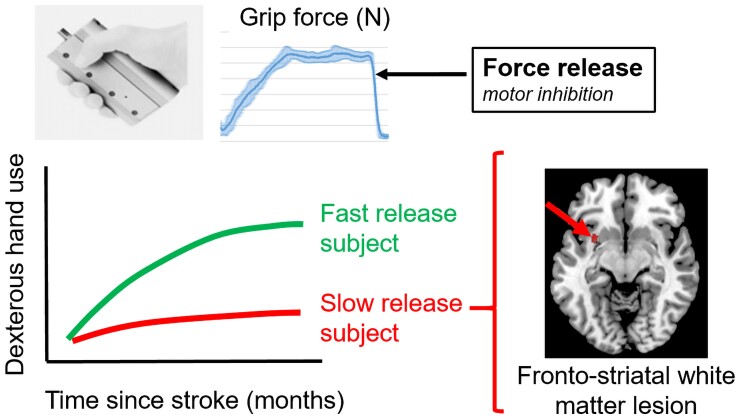

## Introduction

Hand motor impairment, particularly weakness and reduced voluntary movement control, is the most common impairment among stroke survivors,^1^ involving the dexterous use of the hand and leading to difficulties in activities of daily living.^2^ Skilled hand use (grasp and release capability) requires not only the ability to generate sufficient force, but also adequate scaling of forces as well as the ability to inhibit an ongoing force command, for example when releasing a handheld object.^3^ While reduced grip strength is a well-established overall predictor of upper limb motor recovery after stroke,^4^ a broader understanding of how the ability to modulate and release grip force recovers and contributes to dexterous hand use after stroke is lacking. Grip force recordings can inform on motor output (maximal grip force), motor modulation (precision of force control) and motor inhibition (release of grip force) and thus be of value for the development of more efficient personalized post-stroke therapy.

Previous studies have shown that stroke survivors typically produce exaggerated grip forces and have slowed initiation and release of force.^5,6^ Neural inhibition is a common component for both modulation and release of grip force: the former requires gradual^7^ the latter sudden inhibition.^8^ Enhanced precision of grip force modulation may improve post-stroke hand function.^9^ Recently, Pennati *et al*.^10^ showed that recovery of precision grip force control after stroke was related to corticospinal tract (CST) injury, with some additional variance of recovery explained by sensory impairment and initial hand motor impairment. In the chronic phase post-stroke, force release, reflecting motor inhibition functions, may also explain additional variance in the recovery of dexterous hand use beyond that explained by maximal grip force.^11^ However, no study so far has assessed the longitudinal recovery of, and the interplay between, various grip force control measures from the early into the chronic phase post-stroke. And whether these measures explain additional variance in recovery of dexterous hand use remains largely unstudied.

In this longitudinal observational study in stroke survivors, we tested the hypothesis that behavioural measures of motor modulation and inhibition would explain dexterity recovery beyond that explained by measures of motor output alone.^5,10,11^ We utilized grip kinetics to measure four established force control capabilities: (i) maximal isometric power grip force (reflecting motor output),^12,13^ (ii) dynamic precision grip force control (reflecting thumb-index force modulation),^10,14^ (iii) isometric power grip force control (grip force modulation)^5,11^ and (iv) power grip force release (motor inhibition).^11,15^ More precisely, our aims were 2-fold. First, we wanted to investigate how kinetic variables (force generation, modulation and release) contribute to recovery of dexterous hand use, according to Box and Block Test (BBT) (main outcome measure).^16^ Second, since previous behavioural studies have shown some discrepancy in impairment among force control measures,^11^ we investigated the neural correlates of the force control capabilities. We anticipated that the degree of CST injury would be a strong predictor of behavioural measures of motor output, modulation and inhibition.^17^ In addition, we expected that lesions to fronto-striatal networks involved in stopping of actions,^18,19^ would explain additional variance in force modulation and inhibition measures.^8^ Finally, based on previous studies,^20,21^ we also tested whether functional interhemispheric connectivity, measured using resting-state functional MRI, would explain some additional variance in the grip force modulation measures requiring higher level control.^20,21^ Finally, we expected interhemispheric connectivity in the motor cortex (M1) to be related to the performance on more dexterous tasks, such as controlling dynamic forces between the fingertips,^22^ and SMA-M1 intrahemispheric connectivity to be specifically associated with inhibition of an ongoing motor command (release duration).^19,23^

## Materials and methods

### Study design and participants

Eighty-nine patients admitted to a sub-acute in-patient clinic, offering neuro-rehabilitation for persons of working age (18–70 years), were recruited between March 2013 and September 2019. Three assessments were performed: at admission (on average at 3 weeks post-stroke, T1) and at 3 (T2) and 6 (T3) months post-stroke. All patients participated in conventional interdisciplinary rehabilitation.

Inclusion criteria were a first-ever ischaemic or haemorrhagic stroke within 2–6 weeks, with persistent upper extremity hemiparesis. Hemiparesis was verified by clinical examination performed by the admitting physician, using the MRC Manual Muscle Test and the arm and hand items of the National Institute of Health Stroke Scale (NIHSS). Exclusion criteria were (i) inability to comply with or understand instructions, (ii) disorders other than stroke affecting hand function, (iii) a cerebellar lesion and (iv) contraindications for MRI scanning.

All participants provided written informed consent. Speech and language therapists assisted in the recruitment process to ensure that patients with aphasia were able to provide an informed consent. The study was approved by the Regional Ethical Review Board in Stockholm (DNR: 2011/1510-31/3; ClinicalTrials.gov: NCT02878304).

### Assessment of grip force control

1. **Maximal isometric power grip force**
  Maximal grip force was assessed using a digital dynamometer (www.saehan.com). The mean performance of three attempts was recorded. The force of the more affected hand (contralateral to the lesion) was normalized to that of the less affected hand.
2. **Dynamic precision grip force control (referred to as Dexterity-score)**
  Dynamic precision grip was quantified using the Strength-Dexterity Test,^24^ sensitive for the detection of residual impairment of force control between the fingertips after stroke.^10^ This test quantifies the ability to compress a spring with a precision grip (between thumb and index finger) and to dynamically control it in a stable compressed position for about 5 s. This test was repeated using a range of 8 springs in order to identify the longest spring that the patient can compress successfully. The stability of the springs is relative to their respective lengths (free length from 1.80 cm of spring_8 to 4.60 cm of spring_1). The longer springs are thus more unstable and prone to buckling, and require higher demands on precise control of strength and dexterity (spring_1 is the most difficult to compress, and spring_8 is the easiest). Dynamic forces of the index finger and the thumb were recorded using 2 force sensors and analysed off-line using Matlab R2017B (MathWorks, Natick, MA). The derived performance measure used in this study, the Dexterity-score, ranges from 0 to 1, and a higher value represents better performance.^10^
3. **Isometric power grip force control (referred to as Tracking error)**
  Tracking error was derived from a visuomotor force-tracking task^11^ in order to quantify accuracy of isometric power grip force modulation. A decrease in Tracking error indicated better performance. This task has been used to characterize post-stroke force control in the chronic phase.^7^ Tracking error was recorded during the 2 s ramp phase during which the patient scales up force from zero to the target force. Tracking error was quantified by the area (or root mean square) of the absolute difference between the actual force and the target force (5N)^25^ (for further information on set-up, see Supplementary Materials).
4. **Power grip force release (referred to as Release duration)**
  Release duration was derived from the same visuomotor force-tracking task as Tracking error and was computed as the time taken to abruptly reduce the grip force from 75 to 25% of the target force at the end of the hold period. Electromyography (EMG) recordings show that the release phase of the task is accompanied by reduced EMG activity in both forearm flexors and extensors (no active extension present) thus reinforcing that force release relates to motor inhibition (see Supplementary Fig. 1).

### Clinical assessments

Overall dexterous grasp and release capability (i.e. dexterous hand use) was quantified with the BBT and used as the main outcome measure. The BBT comprises a rectangular box, separated by a partition and contains 150 wooden cubes (2.5 × 2.5 cm). The test instruction is to move as many cubes as possible during 60 s, one at a time and with one hand, from one side of the partition to the other.^26^ Normative values for the adult stroke population for the corresponding age group (23–69 years) are 72–86 blocks per minute for females and 68–85 blocks for males.^26^ A minimum detectable change has been estimated to be *n* = 5.5 blocks per minute.^27^

Unimanual arm and hand motor impairment was assessed, using the Fugl-Meyer Assessment for the upper extremity (FMA-UE) (0–60 points).^28,29^ Reflex items were excluded to assess voluntary motor control functions exclusively.^30^

Assessment of cognitive impairment was performed using the Barrow Neurological Institute Screen for Higher Cognitive function (BNIS).^31^ Hand spasticity was assessed using the NeuroFlexor© method (AggeroMedTech.com)^32^ allowing for quantification of the neural component (NC) of the resistance to passive extension of wrist and finger flexor muscles. NC >3.4N (i.e. mean + 3SD in a cohort of *n* = 107 neurologically intact control subjects) was considered as hand spasticity.^33^ Somatosensory impairment (two-point discrimination, 2pD) was assessed with a Disc-Criminator (Dellon-McKinnon). Inability to detect a ≥12 mm separation indicated impairment.

### Magnetic resonance imaging

Anatomical (CST lesion load and voxel-based lesion symptom mapping, VLSM) and functional MRI was used to investigate the structural and functional connectivity (FC) correlates of grip force control recovery. Brain imaging was performed at study inclusion with an Ingenia 3.0 T MR system (www.usa.philips.com) with an 8HR head coil. High-resolution T_1_-weighted anatomical images were acquired using TFE 3D (three-dimensional gradient echo-based sequence): field of view, 250 × 250 × 181 mm; matrix, 228 × 227; slice thickness, 1.2 mm; slice spacing, 0.6 mm and number of slices, 301 (echo time [TE] = 3.5 ms; repetition time [TR] = 7.5 ms). Additionally, T2 fluid-attenuated inversion recovery (FLAIR) images were acquired. Resting-state fMRI data were acquired using a gradient echo-planar sequence (echo time [TE] = 35 ms, flip angle = 90°, voxel size of 1.8 × 1.8 × 4 mm, repetition time [TR] = 3000 ms) sensitive to BOLD contrast. Acquisition time was 6 min and total number of volumes acquired = 160. Patients were instructed to keep their eyes closed, to think about nothing in particular and not to move or fall asleep.

Anatomical T1 images were normalized to the Montreal Neurological Institute template using the SPM12 software package (www.fil.ion.ucl.ac.uk/spm/software/spm12/) Clinical toolbox unified segment-normalize procedure^34^ (non-linear enantiomorphic normalization,^35^ 3-tissue, ‘old segment’, optimizing the normalization of clinical data with focal brain lesions by exploiting information from homologous regions of the non-lesioned hemisphere). Subsequently, the normalization parameters for T1 images were applied to resting-state functional images using the SPM12 tool Old Normalize. Cost function masking was used to avoid distortion of lesion by the normalization procedure, and the images were inspected visually to ensure adequate normalization. Lesion maps were manually drawn on all axial slices of native space T_1_-weighted anatomical images using MRIcron (https://people.cas.sc.edu/rorden/mricron/index.html) by a trained researcher (J.P.) and verified by an experienced neurologist (J-C.B.) who was blinded to all clinical data except the lesioned hemisphere. Localization of lesions was compared and verified to FLAIR images, and binarized lesion maps were created.

#### Weighted CST lesion load

Quantitative lesion maps were calculated to compute the weighted CST lesion load (wCST-LL) using a previously constructed CST template based on regions of interest (ROIs) in the precentral gyri, posterior limb of internal capsule, cerebral peduncle and anteromedial pons.^31,36^

#### Voxel-based lesion symptom mapping

Voxel-based lesion symptom mapping was used to study relationships between force control variables and lesion location in *n* = 74 subjects using the NiiStat toolbox (https://www.nitrc.org/projects/niistat/).^37^ Right-sided lesions were flipped to the left side to enable group analysis. In the analysis, we only included voxels that were classified to belong to lesion tissue in at least 10 patients or more. A whole-brain general linear model (linear regression) permutation method (5000 repetitions) identified voxels where the lesion related separately with each force control measure at T3 (corrected for false discovery rate: *P* < 0.05 FDR). A second analysis used Maximal grip force as a nuisance factor for the other variables (FDR corrected *P* < 0.05).^38^ White matter location of voxels correlating with grip force measures was analysed with Tractotron (http://www.bcblab.com/BCB/Tractotron).

#### Resting-state functional connectivity analysis

Seed-based FC analysis was performed in a subsample of *n* = 57 patients with complete resting-state fMRI data, using the Connectivity toolbox^39^ after conventional pre-processing using SPM 12b software (http://www.fil.ion.ucl.ac.uk/spm/software/spm12). The reason for exclusion was 3-fold. First, data from the first *n* = 16 patients were excluded due to missing voxels caused by errors in the setting of the scanning parameters. Secondly, in *n* = 10 patients, the scanning could not be initiated or not completed due to non-compliance, and thirdly, data from *n* = 7 patients were invalid due to movement artefacts or other artefacts due to large lesions. Image pre-processing included (i) head movement and correction, (ii) co-registration of resting-state fMRI (EPI) images to T_1_-weigted anatomical images, (iii) segmentation (grey matter/white matter/CSF), (iv) normalization using the SPM12 Clinical Toolbox and (v) smoothing (8 mm).

Interhemispheric FC between M1 has been shown to explain a portion of the variance in motor recovery^20,40^ while intrahemispheric FC between M1 and other key motor areas^41^ of the affected hemisphere have been less studied. Here, we calculated both interhemispheric FC between left/right M1 and intrahemispheric contralateral FC between M1 and ROIs^42^ that included the parieto-frontal motor pathways:^43^ supplementary motor area (SMA), ventral and dorsal premotor cortex (vPMC and dPMC, respectively), anterior intraparietal sulcus (aIPS) and rostral cingulate zone (RCZ). The ROIs were spherical with a 10 mm diameter (see Supplementary Fig. 2) Seed-based FC was calculated using the CONN Functional Connectivity Toolbox (web.conn-toolbox.org). The CONN toolbox incorporates the CompCor strategy for reduction of noise of physiological and other sources that take into account the non-homogenous distribution of noise signals in the brain.^44^ For example, voxels close to white matter or large blood vessels show greater BOLD signal noise. Principal components (PCA) were derived from these noise regions and later included as nuisance parameters within the general linear model. EPI images were inspected visually to identify signal drop-out (due to, e.g., the presence of meta-haemoglobin and hemosiderin, i.e. breakdown products from haemorrhagic stroke). A comparison of BOLD signal strength between the lesioned and non-lesioned hemispheres was also performed (Supplementary Fig. 3).

Estimation of head motion parameters and the presence of image outliers (Artifact Detection toolbox: https://www.nitrc.org/projects/artifact_detect) were included as regressors since it has been shown that this strategy improves motion artefact correction when studying FC.^45^ Activation threshold of z-normalized global brain signal was set to 3SD and threshold for rotational and translational head motion was set to 2 mm.^45^ This resulted in mean (±SD) = 10.2 (±11) excluded volumes (out of 160 = 6.4%). White matter and CSF masks were used for partial volume correction. The principal components of signal from white matter and CSF masks were regressed out during the analysis. A temporal band pass filter (0.01–0.08 Hz) was applied covering approximately the range between 10 and 100 s which is standard for resting-state connectivity analyses.^46^ The toolbox computed the average BOLD time series across all the voxels within each ROI.

The beta value reflecting interhemispheric and intrahemispheric FC between each pair of ROIs was extracted for each patient.

### Statistical methods

A Linear Mixed Effect Model with subject ID included as a random effect variable was used to calculate the overall effect of time on each of the four grip force variables. For non-unit specific comparisons of degree of change from baseline (T1) to 3 (T2) and 6 months (T3), standardized effect sizes (ES) were calculated as follows: ES = (mean T2 − mean T1)/(SD at T1) and ES = (mean T3 − mean T1)/(SD at T1), respectively. Bootstrapping resampling with 1000 iterations was performed to obtain 95% confidence intervals.

To explain the variance of recovery (BBT score at 6 months), analysis first involved univariate linear regression to determine the strength of the univariate associations. Secondly, multivariable linear regression analysis was undertaken. A stepwise procedure using forward selection was used. The first regression models included the four kinetic variables and thereafter we added explanatory variables in order of association strength with the dependent variable, i.e. initial BBT, wCST-LL, two-point discrimination, FMA (full and subscale), spasticity as well as SAFE score. The independent variables were carried forward, one by one (from model 1 to 2, to 3 and so forth), in order of univariate association strength (i.e. the highest *R* square). Included variables that did not contribute with a significant *F*-change were discarded. For evaluation of alternative explanatory variables, the analysis procedure was repeated without the strongest predictor identified in the first model. We checked for co-linearity between variables put in to regression models using the Variance Inflation factor (VIF) and all combinations of variables had VIF <3, indicating acceptable co-linearity for multivariate regression.

Change scores (Δ) were calculated by taking the difference between status at 6 months (T3) and 3 weeks (T1). To avoid ceiling effects, the mildly impaired patients (FMA-UE >47 points at T1) were omitted from calculations using Δ scores leaving a sample of *n* = 66.

To enable the use of all data points available, two methods of imputation were performed. In patients that were unable to perform the visuomotor force-tracking task due to paresis, the respective Tracking error and Release duration variables were replaced with worst-case scores obtained at T1. A worst-case score was chosen since a low score on the Visuomotor force-tracking task equals better performance (i.e. shorter release duration and less tracking error). Thus, replacement with zero was not possible. In case of missing data due to loss to follow up or invalid trials, scores were imputed using linear regression with existing data as input variables to the regression equation. Imputation by regression was performed only in case of maximal one data-point missing per case.

We estimated that adequate statistical power, to explain hand use recovery, would require 20 observations for each explanatory variable (based on the previous study *n* = 28^11^). The level of significance was set at 0.05. *P*-values were corrected for multiple comparisons according to the Benjamini-Hochberg procedure (e.g. for 184 *P*-values from univariate correlations presented in Supplementary Table 3).^47^ Analysis was performed using IBM SPSS Statistics 27 (www.ibm.com/products/spss-statistics).

## Results

A total of 89 patients were included in the study, at 25 ± 7 days (mean ± SD) after stroke onset. Main demographics and clinical characteristics of the study cohort are summarized in Table 1. Flowchart of the recruitment process is presented in Supplementary Fig. 4. Among the included patients, 53% had severe initial arm and hand motor impairment (FMA-UE <19), while 21% had moderate and 26% mild impairment (FMA-UE 20–47 and >47, respectively). One patient was ill at T2 and *n* = 5 patients were lost to follow up at T3 because of illness, they could not be reached or had moved to another city. At T1, *n* = 36 were unable to perform the visuomotor force-tracking task due to paresis and obtained a worst-case score. At T2, their number was reduced to *n* = 21, and to *n* = 15 at T3. All available data were included in the analyses (see Statistical methods).

**Table 1:** Demographic and clinical characteristics of the study cohort

Variables		ALL (*n* = 89)
Age (years)		52.3 ± 9.4
Sex	Females	23 (26%)
	Males	66 (74%)
Lesion location	Left	40 (44.9%)
	Right	49 (55.1%)
Stroke type	Ischaemic	61 (68.5%)
	Haemorrhagic	28 (31.4%)
NIH Stroke Scale (Median [IQR])		7 (3–12)
wCST-LL (cc)		3.83 ± 3.7
Neglect^a^		21 (24%)
Aphasia^b^		30 (34%)
Cognitive function (0–50p)^c^		38 (31–44)
Barthel Index (0–100p)		60 (43–100)
Two-point discrimination (absent)^d^		48 (54%)
FMA-UE (0–60p)		23.7 ± 23.0

Data are mean ± SD, median (IQR) or number (%).

NIH Stroke Scale = National Institute of Health Stroke Scale; wCST-LL = weighted Corticospinal Tract Lesion Load; FMA-UE = Fugl-Meyer Assessment for the upper extremity.

a Neglect was assessed with the Baking Tray Task.

b Aphasia was assessed with the neurolinguistic instrument A-NING. An index <4.7 indicates aphasia.

c Cognitive function was assessed with the Barrow Neurological Institute Screen for Higher Cerebral Functions. A score ≥47(50) indicates cognitive impairment.

d Inability to discriminate ≥12 mm indicates impairment.

### Recovery of grip force control measures and their interrelationships

**Figure 1:**
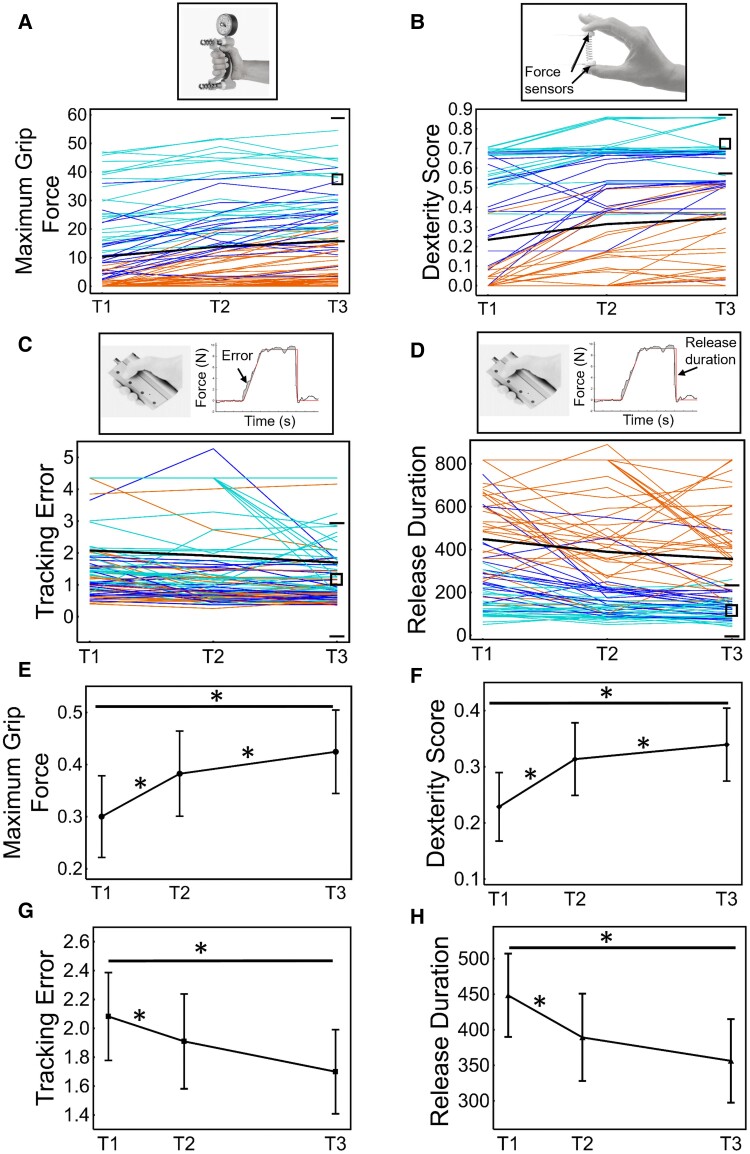
**Recovery patterns of grip force control across the study period (3 weeks to 6 months)**. Upper panel. Individual case profiles of (**A**) Maximal grip force, (**B**) Dexterity-score, (**C**) Tracking error and (**D**) Release duration across the three assessment time points. Colours represent Fugl-Meyer Assessment subgroups according to initial arm and hand motor impairment: severe in orange (≤19 points), moderate in dark blue (20–47 points) and mild in turquoise >47 points). A solid black line represents the group mean. For comparison, the average performance in the ipsilateral hand is depicted on the right of each figure by a black box with upper and lower horizontal bars (mean±2SD) indicating performance in the less affected hand at 6 months. Lower panel (**E–H**). Linear mixed effect model results with estimated marginal means at each time-point with bars showing ±95% CI. * indicate a significant effect of time with *P* ≤ 0.05.

To describe recovery in kinetic measures, we plotted longitudinal data which showed variable individual but similar group-level change over time (Fig. 1A–D and Supplementary Fig. 5, see Supplementary Materials for statistics on recovery). The proportion of patients that did not recover force control to the level of the less affected hand markedly varied between measures (range 17.2–70.4%, Fig. 1A–D and Supplementary Table 1).

At each time-point, grip force control measures correlated with each other (*r*_abs_ range = 0.438 to 0.926, *P* < 0.001 FDR) while associations between change scores were less strongly associated (*r*_abs_ range = 0.120–0.520, *P* = NS to *P* < 0.001 FDR) (for correlation statistics regarding interrelationship between force control measures, see Supplementary Table 2). Change in Dexterity-score correlated with change in Maximal grip force only (*r* = 0.52, *P* < 0.001 FDR) and change in Tracking error only correlated with change in Release duration (*r* = 0.355, *P* < 0.001 FDR).

### Force control measures and dexterous hand use

Four analyses were used to test the main hypothesis that motor modulation and inhibition measures would explain additional BBT recovery beyond that explained by motor output measures.

First, univariate correlations between BBT change scores and initial force control measures were strongest for Tracking error (*r* = −0.478, *P* < 0.001 FDR) followed by Release duration (*r* = −0.404, *P* < 0.001 FDR), while non-significant for Maximal grip force and Dexterity-score (for correlation statistics regarding BBT and grip force variables, see Supplementary Table 3).

Second, a partial correlation analysis using initial BBT as a control variable, showed that among the four initial grip force measures, Release duration at 3 weeks best explained BBT at 6 months (partial *R* = −0.592, *P* < 0.001). Release duration was followed by Tracking error and Dexterity-score in explaining variance, while Maximal grip force did not reach statistical significance after correction for multiple comparisons (Supplementary Table 3).

Third, a multivariable linear regression analysis was then performed with the four kinetic variables to explain variance of BBT score at 6 months while controlling for initial BBT (Table 2). Again, Release duration was the strongest explanatory variable and remained significant even when including other explanatory variables (initial two-point discrimination, wCST-LL, hand spasticity and cognitive impairment one at a time, see also Supplementary Table 4), showing that Release duration explained some unique variance in BBT recovery. This finding was confirmed when including Maximal grip force and force release in the same model, which cancelled the effect of Maximal grip force but not Release duration (models 1 and 2, Supplementary Table 4).

**Table 2:** Multivariable Linear Regression models predicting dexterous hand use (BBT score) at 6 months

Model	Independent variables (at T1)	Unstandardized *B*	Coefficient Std. Error	*R* ^2^ change	Sig.
	(Constant)	9.70	1.93		<0.001
	BBT at T1	0.53	0.16	0.67	0.002
**1** *R* ^2^ = 0.70 (0.69)	and Maximal grip force	27.32	9.47	0.03	0.005
	(Constant)	8.69	1.85		<0.001
	BBT at T1	0.49	0.12	0.67	<0.001
**2** *R* ^2^ = 0.73 (0.72)	and Dexterity-score	41.92	9.26	0.07	<0.001
	(Constant)	24.79	3.13		<0.001
	BBT at T1	0.81	0.07	0.67	<0.001
**3** *R* ^2^ = 0.75 (0.74)	and Tracking error	−5.45	1.05	0.08	<0.001
	(Constant)	38.93	4.45		<0.001
	BBT at T1	0.51	0.09	0.67	<0.001
**4** *R* ^2^ = 0.78 (0.78)	and Release duration	−0.05	0.01	0.11	<0.001
Best fitting model:					
	(Constant)	12.99	5.02		0.011
	SAFE score	6.01	1.31	0.73	<0.001
**5** *R* ^2^ = 0.83 (0.82)	and BBT at T1	0.47	0.07	0.09	<0.001
*R* ^2^ = 0.84 (0.83)	and Release Duration	−0.02	0.01	0.02	0.002

BBT = Box and Block Test.

Model 1–4 corresponds to prediction by two independent variables: BBT at T1 paired with each grip force control measure, one at a time. Model 5 corresponds to prediction based on the independent variables with the strongest univariate associations, entered one at a time using a stepwise forward selection strategy. Unstandardized beta (*B*) expresses the slope of the regression line, i.e. with each unit change in the independent variable, the dependent variable will change with *B*. Coefficient Std. Error represents the standard deviation of the coefficient (*B*) and informs about the precision of the estimate and *R*^2^ (adjusted *R*^2^ in parentheses) indicates the proportion of variance explained by the model.

Fourth, using all clinical, CST injury and kinetic variables, we identified the best fitting model explaining variance of BBT at 6 months. It included SAFE score, initial BBT score and Release duration at 3 weeks, explaining 84% of the variance in BBT at 6 months (Table 2). In this model, Release duration accounted for a small but significant part of the variance, namely 2% (Table 2).

### Neural correlates of force control measures

As expected, CST integrity (wCST-LL) was a strong explanatory variable of all force control measures at 6 months (Supplementary Table 5). The strongest association was observed with Dexterity-score at T3 (*r* = −0.673, *P* < 0.001 FDR). The associations were slightly lower for Release duration (*r* = 0.647, *P* < 0.001 FDR), Maximal grip force (*r* = −0.644, *P* < 0.001 FDR) and Tracking error (*r* = 0.614, *P* < 0.001 FDR) (Supplementary Table 5). The only measure of behavioural change (i.e. improvement from T1 to T3) that correlated with wCST-LL was the Dexterity-score (*r* = −0.326, *P* = 0.003 FDR).

**Figure 2:**
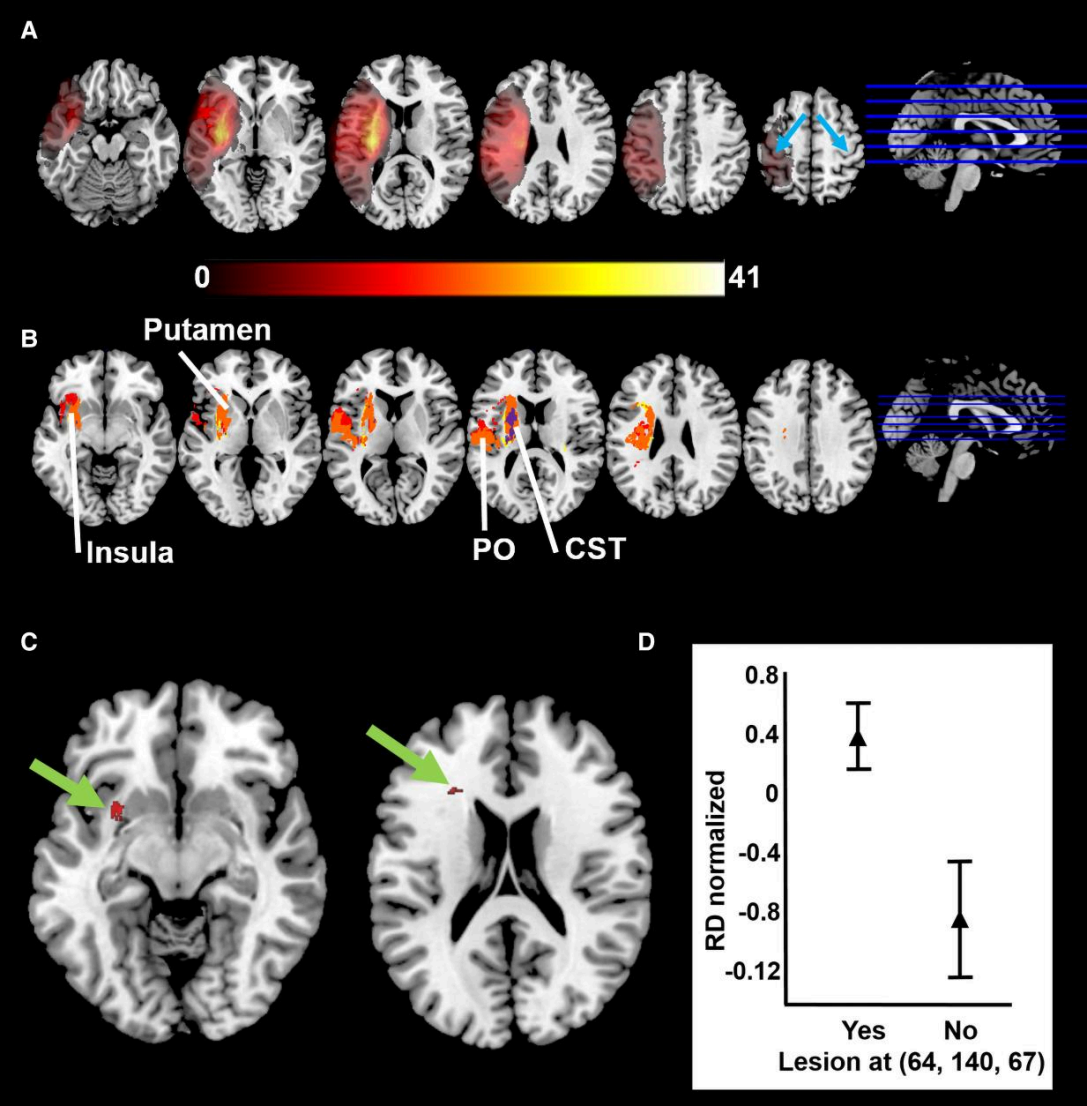
**Lesion distribution of the studied cohort and voxel-based lesion symptom mapping (VLSM) results**. (**A**) Overlapping lesion maps of the cohort (*n* = 74). The most common lesion site was the striato-capsular region including the internal capsule, followed by cortical areas including (but not limited to) the primary motor and somatosensory cortex (hand-knob indicated by blue arrows in the right-most section). Colour code: degree of lesion overlap in 0–41 patients (yellow indicates high overlap). (**B**) Voxel-based lesion symptom mapping (VLSM) showing lesioned voxels relating to force control variables. Blue = Maximal grip force; Red = Tracking error; Yellow = Release duration; Orange = common to Tracking error and Release duration; Violet = common to Maximal grip force, Tracking error and Release duration. Extent of lesion to the corticospinal tract (CST; violet voxels in internal capsule) predicted performance in each variable of force control (Dexterity-score not shown). No unique voxels were found for Maximal grip force. Tracking error (red) showed some unique voxels in insular cortex and parietal opercular (PO) region. Release duration (yellow) showed some unique voxels in white matter extending more anteriorly to CST. Note the common extended pattern of voxels relating to both Tracking error and Release duration (orange) including insular cortex, parietal operculum, external capsule, fronto-parietal white matter and putamen. (**C**) VLSM analysis including Maximal grip force as a nuisance regressor revealed significant voxels within the fronto-parietal white matter (green arrows) that specifically predicted increased force-tracking error and longer release duration; (**D**) Normalized Release duration (at T3) as a function of absence (No) or presence (Yes) of lesions to fronto-parietal white matter shown to the left in (**C**) (Student *T*-test: *T* = 5.46, *P* < 0.001). Higher normalized Release duration (RD) reflects longer release duration.

The distribution of lesions in the study cohort is displayed in Fig. 2A. Voxel-based lesion symptom mapping showed that lesions to the CST (corona radiata, internal capsule) and the striato-capsular region were associated with poorer force control in each variable (Fig. 2B). Weak Maximal grip force was related to lesions limited to the internal capsule (depicted in violet in Fig. 2B). Poor Dexterity-score correlated to tissue damage in similar areas as with Maximal grip force (not shown). In contrast, a generally overlapping and more extended pattern of lesioned voxels was related to larger Tracking error and longer Release duration (shown in orange in Fig. 2B). This involved the insular cortex, parietal operculum (including SII, the secondary somatosensory area), external capsule and putamen. As hypothesized, when including Maximal grip force as a nuisance regressor, lesion to the fronto-striatal pathways (Fig. 2C) was specifically associated with increased Tracking error and longer Release duration. Figure 2D shows Release duration in patients with or without lesion to fronto-striatal pathways. Tractotron analysis of identified voxels (Fig. 2C) confirmed overlap with fronto-striatal tract and with anterior thalamic projections, CST, and frontal aslant tract. Using multiple regression, we found that lesion to fronto-striatal white matter (voxels shown in Fig. 2C) explained an additional 5% of variance in Release duration beyond that explained by wCST-LL alone (43%).

Finally, resting-state FC at T1 did not correlate with any of the force control measures at T3, when corrected for multiple comparisons (Supplementary Table 5). However, there was a tendency for association between interhemispheric M1 FC at T1 and a decrease in Release duration over time (change scores) (*r* = −0.258, *P* = 0.052 FDR). Moreover, intrahemispheric M1-SMA FC at T3 showed a statistically significant association with Tracking error at 6 months (*r* = −0.435, *P* = 0.001 FDR) and a tendency for association with Release duration at T3 (*r* = −0.363, *P* = 0.008 FDR) (significance level after Benjamini-Hochberg correction: *P* ≤ 0.001).

## Discussion

This study presents recovery data on the capability to generate, precisely modulate and release grip force after stroke, providing novel information on maximal motor output and on modulatory and inhibitory cerebral functions. Despite similarities in recovery over time, some differences between these force control capabilities were detected. Importantly, force release, a little studied aspect of force control, was a particularly strong explanatory variable of dexterous hand use at 6 months post-stroke, as assessed with the BBT. This variable, which reflects motor inhibition, captured a unique source of variance in dexterous hand use, not captured by metrics of force output. Although the degree of CST injury correlated to outcome in all force control variables, lesions involving the fronto-striatal tracts correlated only with impairment in force modulation and release, and explained additional variance in force release beyond that explained by CST injury.

### Force release and recovery of dexterous hand use

Force release times remained substantially prolonged at 6 months, with an overall mean of 356 ms, about three times as long compared to that in the less affected hand (Supplementary Table 1). Release duration of the less affected hand (∼114 ms) was close to values found in healthy control subjects (∼90 ms) using an identical paradigm.^11^ The release duration measures the time taken to reduce the force and does not depend on time of release onset, as previously shown in stroke patients.^11^ Several results indicate that force release (Release duration) captures a unique part of the variance regarding outcome (status at 6 months) and recovery (6-month status minus initial scores) of dexterous hand use as assessed by the BBT. First, Release duration explained the largest amount of unique variance in BBT outcome when taking initial BBT score into account (partial *R* = −0.592 versus Maximal grip force *R* = 0.318). Similarly, when comparing patients with complete versus incomplete recovery in each variable, force release again had the strongest predictive effect on BBT outcome. Second, the effect of Release duration remained significant after controlling for other covariates (sensory function, hand spasticity and CST integrity) in the multivariable linear regression model (Supplementary Table 4). Third, linear regression analysis showed that Release duration remained a significant factor explaining BBT recovery even when controlling for Maximal grip force. In contrast, Maximal grip force did not remain significant in the model when controlling for Release duration. Taken together, therefore, our results indicate that force release, which reflects the brain’s capability to inhibit motor commands,^11,48^ explains a significant and complementary part of the variance in recovery of dexterous hand use after stroke.

Thus, prolonged force release may hamper object handling, such as grasp and release, and could be influential in recovery in dexterous hand use. Previous findings showed that weakness in wrist and finger extensor muscles is a major contributor to impaired hand function.^49,50^ Some studies have reported that grasping^51^ and reaching^52^ also require selective inhibition. At grasp onset, poor relaxation of flexor muscles may interfere with hand opening by not allowing full extension of the wrist, fingers and thumb.^50,53^ Prolonged force release may also interfere with grasp configuration by hindering opening of fingers during quick repositioning of the fingers during prehension.^54,55^ Furthermore, the ability to carefully put down and release an object is also hampered by impaired flexor muscle inhibition.^50,56,57^ Our results showed that quantitative measures of hand spasticity only explained a small portion of variance in BBT scores. The univariate association between hand spasticity and recovery of dexterous hand use did not remain statistically significant when adding force release to the model, indicating partly shared variance between these variables. However, the amount of variance in BBT explained by force release was not affected by the inclusion of hand spasticity measures in the same model, indicating that prolonged force release is not an exchangeable proxy for spasticity, as previously reported.^58^

A combined assessment of muscle strength in finger extension and shoulder abduction (rated according to the MRC/SAFE score) is the first step in the Prep prediction algorithm.^59–61^ In the present study, a modified SAFE score (based on FMA-UE) did explain most of the variance accounted for by force release (Table 2). Only a small but statistically significant amount of variance explained by Release duration remained when adding SAFE score to the model. The SAFE-score, a strong overall predictor of hand motor recovery,^62^ thus seems to capture part of the variance explained by Release duration.

It has previously been shown^9^ that improved tracking accuracy, after force-tracking training in patients with chronic stroke, correlates with better grasp and release capacity (according to BBT). We here show that early force modulation is associated with recovery of dexterous hand use over time, and that this association is independent of Maximal grip force, but not of force release (Supplementary Table 1). This result suggests a common behavioural component underlying force-tracking accuracy and force release. We interpret this commonality as a mutual dependence on a common neural substrate: neural inhibition, finely regulated during force modulation, strongly and physically activated for force release, to, respectively, shape or suppress motoneuronal excitation. These two aspects of force control seem to be relevant for recovery of functional hand use after stroke.

### Neural correlates of force control

Results from two independent analyses (CST lesion load and VLSM) showed that lesion to the CST and its degree were related to impaired grip force control according to all four force control measures. As previously reported, greater CST injury was related to impaired maximal force generation^63,64^ and precision grip,^10,65,66^ but also with graded modulation and brisk release of force (Supplementary Table 3). This indicates that an intact CST is crucial for the ability to generate, modulate and stop active grasping. This is consistent with the many actions relayed by the CST,^67^ and in particular with its most direct actions: monosynaptic excitation and disynaptic inhibition of motoneurons,^68,69^ both shown to be involved and regulated in grip force modulation.^7^ Furthermore, the CST is itself under inhibitory control: previous studies using transcranial magnetic stimulation (TMS) have shown that intracortical inhibition contributes to adjusting CST excitability.^70^ This allows for a selective activation of CST projections from M1,^55^ crucial for dexterous control of hand and finger movements.^67^ Other TMS studies have found that increased intracortical inhibition may play a role in suppressing CST excitability during force release.^71,72^ Our results show that CST integrity after stroke is crucial for both excitation and inhibition, enabling voluntary modulation and release of grip force.

Complementary to CST lesion load, VLSM analysis indicated that other areas are related to deficient modulation and release of grip force. Poor performance in Tracking error and Release duration was associated with lesions to secondary somatosensory processing areas (parietal operculum and insular cortex),^73^ as well as to the putamen and fronto-striatal tracts. Parietal areas have been implicated in control of grip force^74–76^ as well as in action control^77^ and presumably reflect higher demands of sensorimotor integration during dexterous visuomotor control. When including Maximal grip force as a nuisance regressor, lesion of voxels within fronto-striatal pathways, rostral to the CST, specifically predicted prolonged Release duration and poor precision in the force-tracking task. The anatomical localization suggests a contribution of cortico-striatal pathways for motor inhibition, likely to be important for both force modulation (Tracking error) and release. White matter tracts connecting the posterior medial frontal cortex and the subthalamic nucleus have been identified as important for stopping an ongoing action.^78^ Cortico-subthalamic connection strength in ageing has also been found to predict stopping performance.^79^ In a functional MRI study in healthy subjects, activations in inferior frontal gyrus, pre-supplementary motor area and subthalamic nucleus occurred with motor inhibition^80^ and the strength of brain activation predicted individual variability in stopping performance. Despite a likely involvement of these structures and their white matter connections in motor inhibition, other cortical and subcortical regions may also contribute to the modulation of CST excitability, such as the reticular formation.^81^

Contrary to our hypothesis, none of the resting-state FC measures at T1 were significantly associated with any of the force control measures at 3 weeks or at 6 months (i.e. *P* > 0.001, Benjamini-Hochberg corrected). We only saw a tendency for association between interhemispheric M1-M1 connectivity at T1 and a decrease in Release duration over time (change scores) (Supplementary Table 5). Consequently, our results did not corroborate the previously reported value of interhemispheric M1-M1 connectivity for the explanation of hand motor recovery in the sub-acute post-stroke phase.^82^ However, we have recently shown that interhemispheric M1-M1 connectivity was the second strongest explanatory variable of hand motor recovery according to the FMA Hand subscale and FMA total, outperforming other known predictors such as sensory function and CST integrity.^62^ This suggests that interhemispheric M1-M1 connectivity may account for some variance in hand motor recovery assessed by more gross outcome measures,^62^ but not for kinetic measures of grasping.

Finally, our expectation about the role of SMA was supported by a statistically significant association between ipsilesional M1-SMA intrahemispheric connectivity and precision modulation of grip force, and an (uncorrected) association with force release. These findings, together with our results regarding the association between integrity of cortico-striatal white matter tract and motor inhibition, support a key role of SMA for stopping of motor behaviour after stroke.^19,78^

### Limitations

This study has some limitations. First, the probabilistic template used in developing the weighted CST lesion load measure identified the CST as exclusively originating in the M1 hand area. We therefore cannot control for the contribution of other CST fibres originating outside M1.^67^ Although not including all CST projections, this template has proven to detect clinically valuable information on CST integrity.^36,58^ A similar template used in other studies has also given similar correlations with post-stroke motor impairment.^83^ Second, the FC analyses failed to invalidate the null-hypothesis. Although signal drop-out due to the presence of meta-haemoglobin and hemosiderin,^84^ i.e. breakdown products from haemorrhagic stroke, may have affected our data, a comparison of BOLD signal strength revealed no significant differences between the lesioned and non-lesioned hemispheres (Supplementary Fig. 2). Third, stroke patients may adopt different grasping strategies while performing the BBT.^85^ This was not assessed in this study. However, longitudinal studies on stroke recovery are required to investigate whether altered grasping strategies could help to explain variance in recovery. Lastly, some patients could not perform the visuomotor force-tracking task and therefore obtained a worst-case score. These floor values may have decreased the variance in the sample and perturbed the analysis. However, imputing data from these patients enabled us to study force control recovery in a larger cohort, and importantly, to include patients with initially severe hand motor impairment. Exclusion of these patients did not alter the main findings, either in behavioural terms or in terms of explanation (multivariable regression analysis of recovery) or in relation to the neural substrates. To note the relatively young age of this study cohort (52.3 ± 9.4 years) which limits generalization of these findings to stroke survivors of higher age.

## Conclusion

This longitudinal study revealed persistent impairment of grip force generation, modulation and release at 6 months after stroke. Partial recovery of grip function was related to lesion site: the degree of CST injury was a major determinant of recovery of grip force control, including recovery of force modulation and release. Lesion to pathways specifically involved in motor inhibition explained additional variance in recovery of force modulation and force release. At the behavioural level, poor initial force control capacity predicted limited dexterous hand use at 6 months. This association was particularly strong for force release, a probe of motor inhibition and a less studied aspect of force control. Importantly, the association of force release with post-stroke hand use remained significant even after including the clinical SAFE score in the explanatory model. This novel finding highlights the importance of motor inhibition for hand motor recovery after stroke. Taken together, our findings open new avenues for targeted rehabilitation programmes aimed at improving grasp and release capacity for dexterous hand use after stroke.

## Supplementary Material

fcac241_Supplementary_Data

## Data Availability

Anonymized data will be shared by request from any qualified investigator within the EU.

## Abbreviations

2pD =: two-point discrimination
aIPS =: anterior intraparietal sulcus
BNIS =: Barrow Neurological Institute Screen for Higher Cognitive function
BBT =: Box and Block Test
CST =: corticospinal tract
dPMC =: dorsal premotor cortex
FC =: functional connectivity
FMA-UE =: Fugl-Meyer Assessment for the upper extremity
M1 =: primary motor cortex
NC =: neural component
NIHSS =: National Institute of Health Stroke Scale
RCZ =: rostral cingulate zone
ROI =: region of interest
SMA =: supplementary motor area
vPMC =: ventral premotor cortex
VLSM =: voxel-based lesion symptom mapping
wCST-LL =: weighted corticospinal tract lesion load
