# Supplementary material for: Motor inhibition and its contribution to recovery of dexterous hand use after stroke
Source: Brain Commun. 2022 Sep 23;4(5):fcac241. doi: 10.1093/braincomms/fcac241 (PMC9562786; doi:10.1093/braincomms/fcac241)
Supplement: fcac241_Supplementary_Data [file fcac241_supplementary_data.docx]

Supplementary materials

# Methods

## Assessment of grip force control

During the visuomotor force tracking task, the patient was seated in a chair with full back support with the hand and forearm resting in the lap or on a table in a mid-prone position. A manipulandum consisting of two levers, acting on a force transducer, was positioned in the palm of the hand. The patient exerted isometric grip force displayed in real-time by a cursor on a 12-inch computer screen. All patients were instructed to follow, as precisely as possible, a target ramp-hold-and-release force trajectory with the cursor and to release the force rapidly at the end of the hold phase. The task comprised 12 blocks, each consisting of 4 ramp-hold-and-release target force trajectories, similar to a paradigm used previously.^7^ Two different target (hold) forces were alternated between blocks, one absolute (5N) and one relative (10% of each patient’s maximal voluntary grip force). Of note, tracking error has been found to be negatively and exponentially related to absolute force level in patients with stroke, in both the ipsilateral and contralateral hands, and in neurologically intact control subjects.^7^ We therefore expected the 5N target force level to provide a sensitive error measure in patients within the whole range of hand motor impairment and selected the 5N (and not the relative force level) for further analysis. Force output was amplified and then sampled at 1 kHz by a CED Micro1401 running Spike2 (Cambridge Electronic Design®) and pre-processed using Matlab R2018B (MathWorks, Natick, MA). Tracking error were averaged across (5N) blocks.


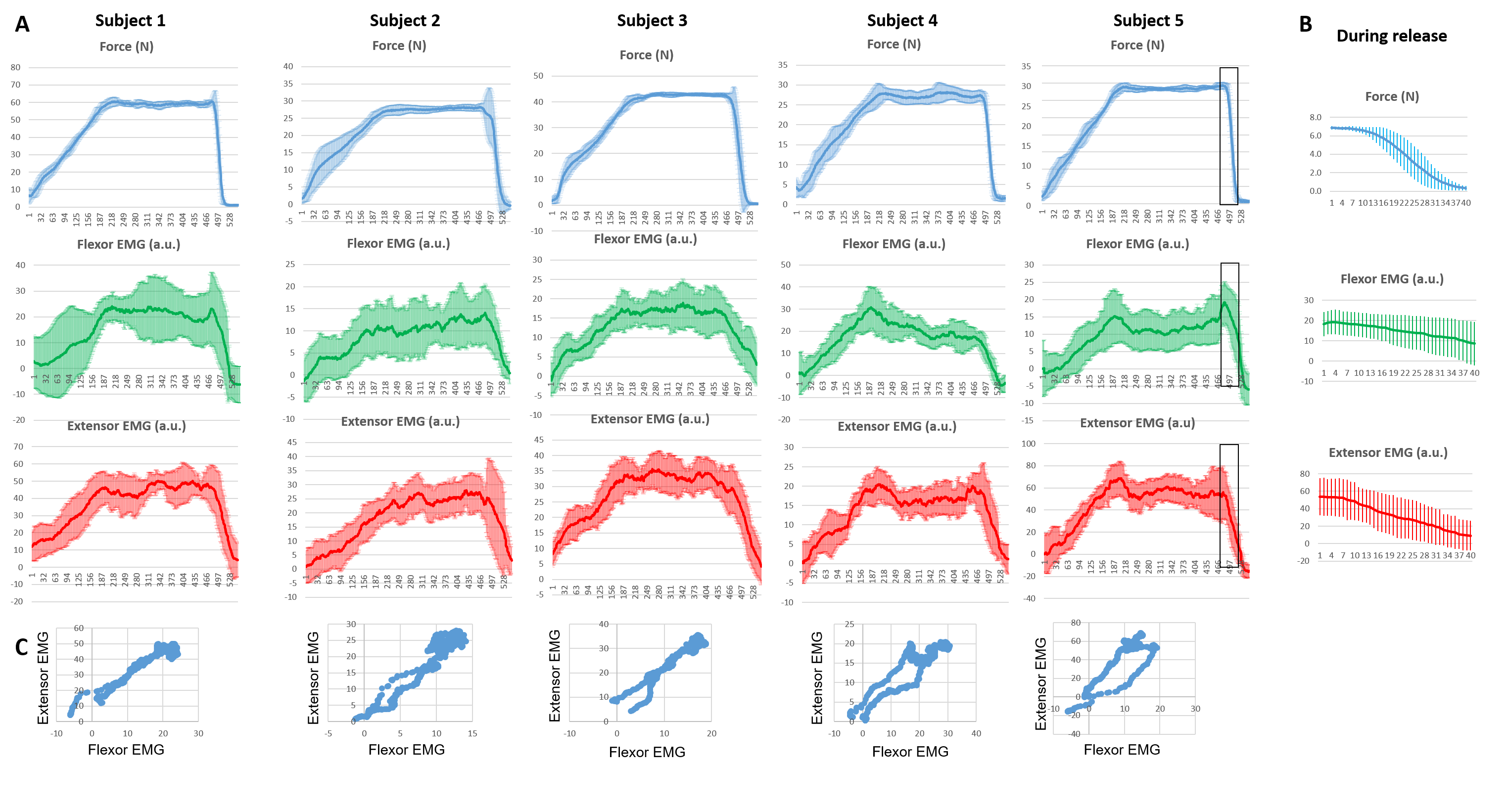


**Supplementary Figure 1**

Power grip force and flexor and extensor electromyography (EMG) recordings during visuomotor force-tracking task in five healthy subjects. A) First row shows mean force of 10 trials (blue line) with ±SD shown in light blue shading (each time bin represents 100ms, i.e., 500 time bins correspond to 5s trial duration). Second row shows mean±SD flexor carpi radialis (FCR) EMG activity (in green) and third row shows mean±SD extensor digitorum communis (EDC) EMG activity (in red). Note that flexor and extensor activity show co-activation during force generation, and both show co-deactivation during force release. B) A zoom in on 400ms of the the release phase (indicated by the open black columns in A) in subject 5 shows a clear correspondence between decreasing force, i.e. grip release, and decreasing EMG activity in both flexor and extensor muscles. C) Each subject’s FCR and EDC EMG traces correlated strongly indicating task-related co-activation of flexor and extensor muscles (Pearson correlation tests: R=0.87 to 0.96, P<0.001).

Furthermore, we have previously provided computational evidence consistent with these EMG recordings, that a reduction of task-related motor inhibition is sufficient to explain an increased release duration, as empirically found in patients with schizophrenia in the same behavioral paradigm (Térémetz et al. 2014).


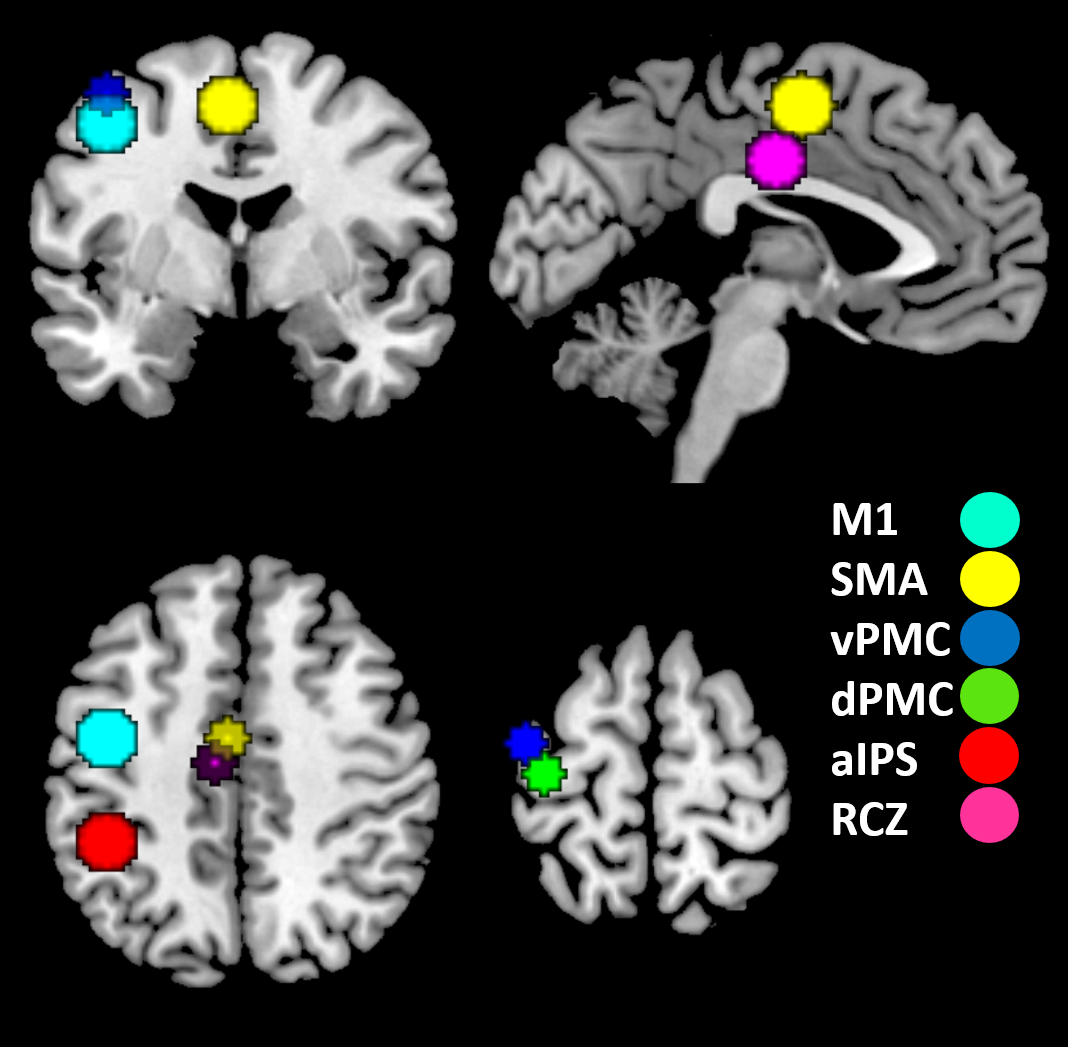


**Supplementary Figure 2**

Functional connectivity (FC) analyses between left/right M1 and intrahemispheric contralateral FC between the primary motor cortex (M1), depicted in turquoise, and other regions within the sensorimotor network (identified in meta-analysis by Rehme et al., 2012) according to were calculated including the following regions of interest (ROIs): supplementary motor area (SMA) in yellow, ventral premotor cortex (vPMC) in blue, dorsal premotor cortex (dPMC) in green, anterior intraparietal sulcus (aIPS) in red and rostral cingulate zone (RCZ) in pink.

References:

Térémetz M, Amado I, Bendjemaa N, Krebs M-O, Lindberg PG, Maier MA. Deficient grip force control in schizophrenia: behavioral and modeling evidence for altered motor inhibition and motor noise. PLoS ONE 2014, e111853. doi:10.1371/journal.pone.0111853*.*

Rehme AK, Eickhoff SB, Rottschy C, Fink GR, Grefkes C. Activation likelihood estimation meta-analysis of motor-related neural activity after stroke. Neuroimage. 2012 Feb 1;59(3):2771-82


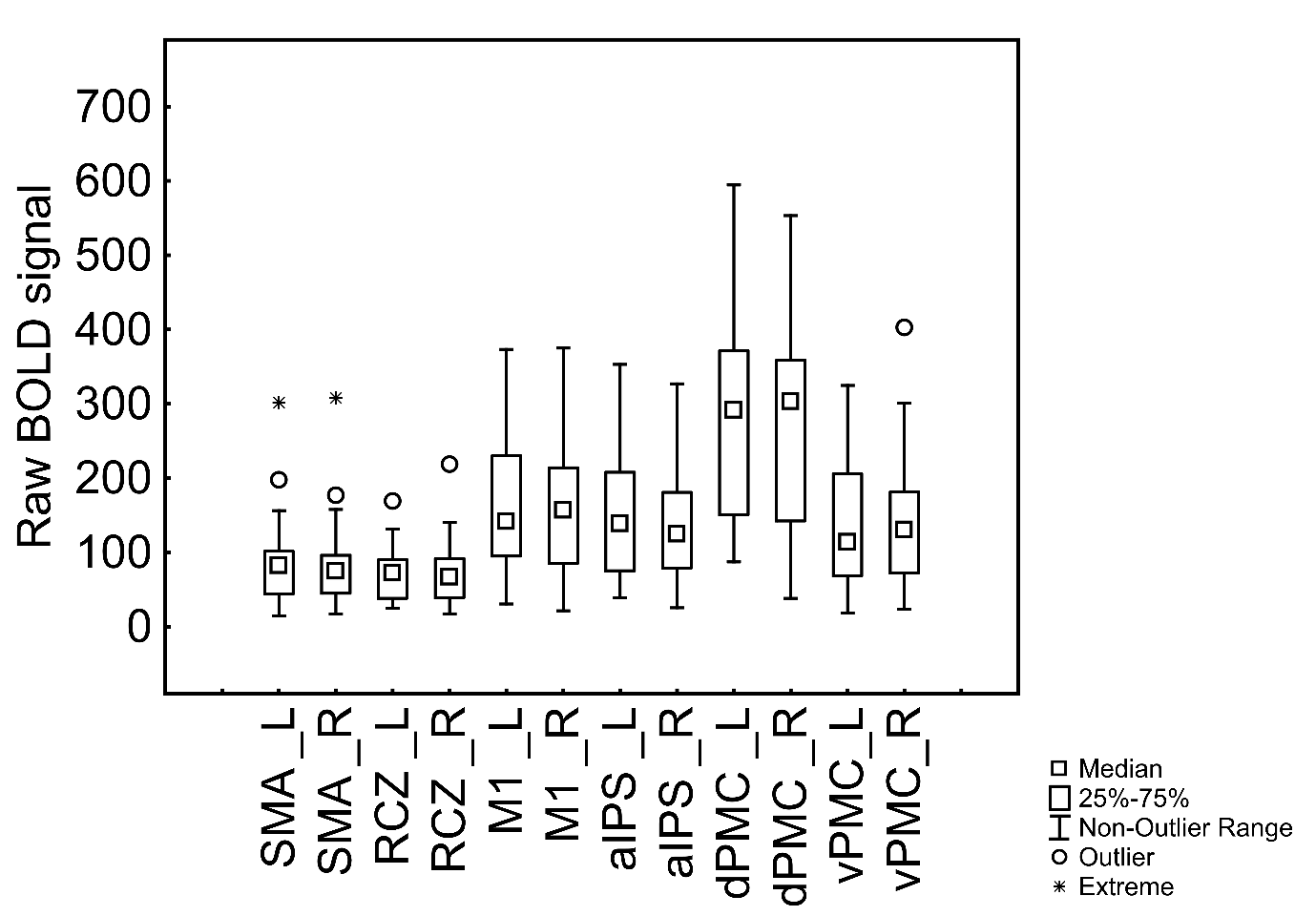


**Supplementary Figure 3**

Box-plots of raw BOLD signal of the respective regions of interest (ROIs). Note: all lesions were flipped to the left hemisphere. Thus, left (L) represents the affected/lesioned side. A comparison of signal strength between pairs of ROIs (L vs R) of the respective hemisphere showed no significant differences (ANOVA: SIDE F=1.43, P=0.24). Abbreviations: supplementary motor area (SMA), rostral cingulate zone (RCZ), primary motor cortex (M1), anterior intraparietal sulcus (aIPS), dorsal premotor cortex (dPMC) and ventral premotor cortex (vPMC).


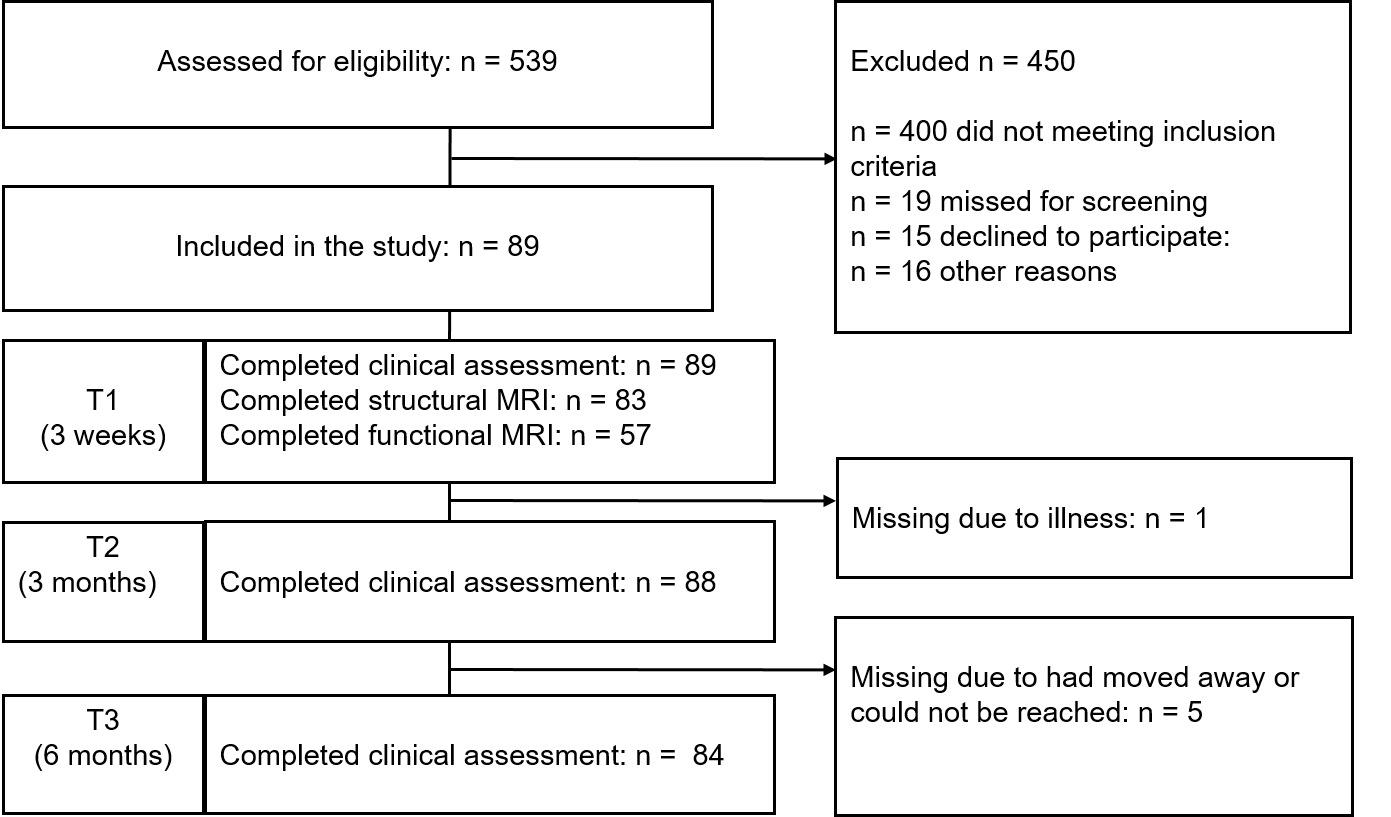


# Supplementary Figure 4 Flowchart of the recruitment process. Assessments were performed at day 25±7 (mean±SD) (T1), day 93±10 (T2) and 188±12 (T3).

# Results

**Recovery of grip force control measures and their interrelationships**

There was a high inter-individual variability in each force control measure at each time-point. Degree of change (recovery) also varied widely (Figures 1 A-D). At group level, there was an overall significant effect of time for each force control measure (Maximal grip force [F_2,88_ = 21.8, p<0.001], Dexterity-score [F_2,86_ =20.2, p<0.001], Tracking error [F_2,88_ =11.6, p<0.001] and Release duration [F_2,88_ =16.5, p<0.001]). Post hoc tests showed a statistically significant change from T1 to T2 in all measures (*P* < 0.004) and for Maximal grip force (EM mean difference [SE] = 0.042[0.01], 95%CI 0.01-0.07) and Dexterity-score (EM mean difference[SE] = 0.026[0.01], 95%CI 0.01-0.04) between T2 and T3 (Fig. 1 E-H). Effect-sizes, reflecting non-unit specific extent of recovery, were similar between measures (range 0.26 to 0.39, Table 2 and Fig. 2).


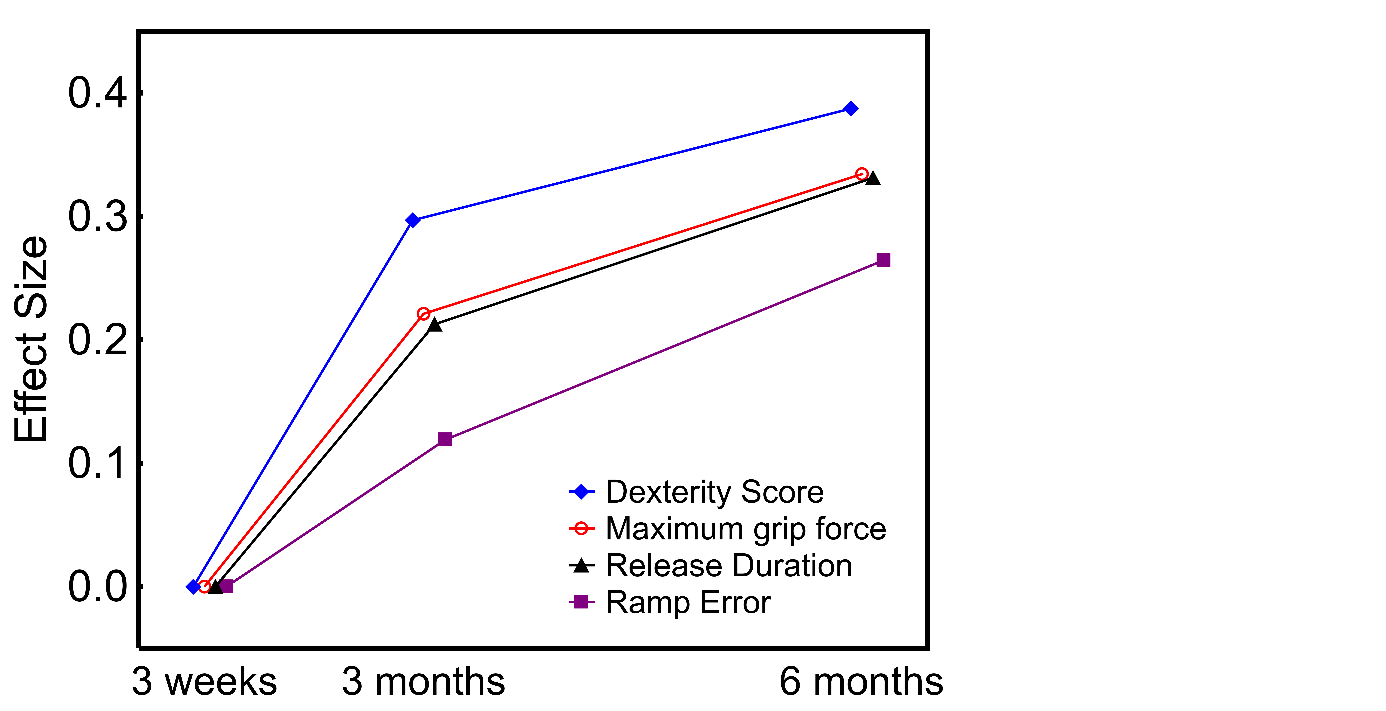


**Supplementary Figure 5** Standardized change in grip force control measures from T1 (3 weeks) to T2 (3 months) and T3 (6 months) post-stroke. Illustration of recovery in force control variables. Performance in each score with standardized effect size (ES) calculated as follows: ES at 3 months = (mean T2-mean T1)/(SD at T1) and ES at 6 months = (mean T3-mean T1)/(SD at T1). Colours represent: Dexterity-score in blue, Maximal grip force in red, Release duration in black and Tracking error in purple.

**Supplementary Table 1** Comparison of Box and Block Test scores between recovery groups.

| One way analysis of variance | | | | |
| --- | --- | --- | --- | --- |
| Variables | Proportion of patients who recovered to the level of the less affected hand* | Box and Block Test  mean±SD | Eta squared | Sig. (P) |
| Maximal grip force | YES (44.9%) | 48.9±15.7 | 0.66 | <0.001 |
|  | NO (55.1%) | 7.8±10.3 |  |  |
| Dexterity Score | YES ( 29.6%) | 55.5±10.6 | 0.50 | <0.001 |
|  | NO (70.4%) | 15.6±20.0 |  |  |
| Tracking Error | YES (82.8%) | 31.6±24.4 | 0.23 | <0.001 |
|  | NO (17.2%) | 0.1±0.26 |  |  |
| Release Duration | YES (54%) | 46.1±16.5 | 0.70 | <0.001 |
|  | NO (46.0%) | 4.0±10.3 |  |  |
| *Positive recovery (Yes) was defined as equalling the performance of the less affected hand at 6 months (mean±2SD). | | | | |

**Force control measures and dexterous hand use**

At each time-point, Box and Block Test (BBT) scores correlated significantly with each of the 4 force control measures, and the strongest association was with Maximal grip force at T2 (*r* = 0.93, *P* < 0.0001) and the lowest with Tracking error at T1 (*r* = 0.424, *P* < 0.001) (Supplementary Table 3). Correlations between BBT at 6 months and initial grip force control measures at T1 was strongest for force release (*r* = -0.837, *P* < 0.001) and lowest for Tracking error (*r* = -0.597, p<0.001). Correlations between BBT change scores and initial force control measures were strongest for Tracking error (*r* = -0.478, *P* < 0.001) followed by Release duration (*r* = -0.404, *P* < 0.001) while non-significant for Maximal grip force and Dexterity-score (Supplementary Table 3).

**Supplementary Table 2** Interrelationship between grip force control measures and associations between grip force control measures and Box and Block Test scores.

| Full  correlations | | Max. grip force | | | | | Dexterity score | | | | | Tracking error | | | | | Release duration | | | |
| --- | --- | --- | --- | --- | --- | --- | --- | --- | --- | --- | --- | --- | --- | --- | --- | --- | --- | --- | --- | --- |
|  | | T1 | T2 | T3 | Δ | T1 | | T2 | T3 | Δ | T1 | | T2 | T3 | Δ | T1 | | T2 | T3 | Δ |
| Max. grip force | T1 |  |  |  |  |  | |  |  |  |  | |  |  |  |  | |  |  |  |
|  | T2 | 0.94* |  |  |  |  | |  |  |  |  | |  |  |  |  | |  |  |  |
|  | T3 | 0.89* | 0.96* |  |  |  | |  |  |  |  | |  |  |  |  | |  |  |  |
|  | Δ | -0.18 | 0.09 | 0.28 |  |  | |  |  |  |  | |  |  |  |  | |  |  |  |
| Dexterity score | T1 | **0.89*** | 0.84* | 0.84* | 0.00 |  | |  |  |  |  | |  |  |  |  | |  |  |  |
|  | T2 | 0.86* | **0.90*** | 0.93* | 0.220 | 0.90* | |  |  |  |  | |  |  |  |  | |  |  |  |
|  | T3 | 0.83* | 0.88* | **0.93*** | 0.28 | 0.85* | | 0.97* |  |  |  | |  |  |  |  | |  |  |  |
|  | Δ | -0.02 | 0.16 | 0.25 | **0.52*** | -0.17 | | 0.25 | 0.38* |  |  | |  |  |  |  | |  |  |  |
| Tracking error | T1 | **-0.44*** | -0.54* | -0.59* | -0.36* | **-0.44*** | | -0.60* | -0.62* | -0.39* |  | |  |  |  |  | |  |  |  |
|  | T2 | -0.46* | **-0.55*** | -0.59* | -0.34* | -0.43* | | **-0.61*** | -0.62* | -0.39* | 0.95* | |  |  |  |  | |  |  |  |
|  | T3 | -0.44* | -0.51* | **-0.55*** | -0.31 | -0.43* | | -0.54* | **-0.56*** | -0.29 | 0.82* | | 0.83* |  |  |  | |  |  |  |
|  | Δ | 0.04 | 0.09 | 0.11 | **0.12** | 0.04 | | 0.14 | 0.13 | **0.18** | -0.367 | | -0.26 | 0.23 |  |  | |  |  |  |
| Release duration | T1 | **-0.77*** | -0.81* | -0.82* | -0.15 | **-0.76*** | | -0.83* | -0.83* | -0.21 | **0.70*** | | 0.68* | 0.63* | -0.18 |  | |  |  |  |
|  | T2 | -0.72* | **-0.80*** | -0.81* | -0.27 | -0.71* | | **-0.85*** | -0.84* | -0.32 | 0.76* | | **0.77*** | 0.69* | -0.17 | 0.89* | |  |  |  |
|  | T3 | -0.67* | -0.75* | **-0.78*** | -0.34 | -0.68* | | -0.78* | **-0.80*** | -0.30 | 0.70* | | 0.68* | **0.75*** | 0.02 | 0.84* | | 0.85* |  |  |
|  | Δ | 0.17 | 0.10 | 0.05 | **-0.28** | 0.12 | | 0.07 | 0.02 | **-0.16** | 0.01 | | 0.00 | 0.22 | **0.36*** | -0.28 | | -0.06 | 0.29 |  |
| BBT | T1 | **0.91*** | 0.87* | 0.82* | -0.13 | **0.84*** | | 0.82* | 0.77* | -0.05 | **-0.42*** | | -0.44* | -0.42* | 0.04 | **-0.75*** | | -0.68* | -0.64* | 0.19 |
|  | T2 | 0.87* | **0.93*** | 0.92* | 0.15 | 0.85* | | **0.94*** | 0.92* | 0.22 | -0.54* | | **-0.55*** | -0.52* | 0.07 | -0.84* | | **-0.81*** | -0.77* | 0.12 |
|  | T3 | **0.82*** | 0.89* | **0.92*** | 0.27 | **0.82*** | | 0.94* | **0.94*** | 0.30 | **-0.60*** | | -0.59* | **-0.55*** | 0.12 | **-0.84*** | | -0.83* | **-0.81*** | 0.06 |
|  | Δ | **0.14** | 0.34 | 0.43* | **0.66*** | **0.26** | | 0.50* | 0.58* | **0.62*** | **-0.48*** | | -0.43* | -0.40* | **0.17** | **-0.40*** | | -0.49* | -0.52* | **-0.20** |

**Abbreviations**: BBT – Box and Block Test. Numbers are Pearson correlation coefficients, * indicate statistical significance after correction for multiple comparisons (P = ≤0.001). Δ = change scores. Note: negative correlations for Tracking error and Release duration, for which smaller values indicate better performance.

**Supplementary Table 3** Interrelationship between grip force control measures and Box and Block Test scores.

| Full correlations | | Max. grip force | Dexterity score | Tracking error | Release duration |
| --- | --- | --- | --- | --- | --- |
|  |  | T1 | T1 | T1 | T1 |
| Box and Block Test (BBT) | T1 | 0.906* | 0.841* | -0.424* | **-0.751*** |
|  | T2 | 0.871* | 0.845* | -0.536* | -0.837* |
|  | T3 | 0.815* | 0.825* | -0.597* | -0.837* |
|  | Δ | 0.140 | 0.259 | -0.478* | -0.404* |
| Partial correlations^a^ |  | | | | |
| Box and Block Test (BBT)  (Control variable: BBT at T1) | T3 | 0.318 | 0.445* | -0.494* | -0.592* |

**Abbreviations**: BBT – Box and Block Test. Numbers are Pearson correlation coefficients, * indicate statistical significance after correction for multiple comparisons (p ≤0.001). Δ = change scores. a Partial correlation was calculated including BBT at T1 as a control variable. Note: negative correlations for Tracking error and Release duration, for which smaller values indicate better performance.

**Supplementary Table 4** Multivariable Linear Regression models explaining variance of dexterous hand use (BBT score) at 6 months, controlling for sensory function, CST lesion load, hand spasticity and Fugl-Meyer Assessment

| Model | Independent variables (at T1) | Unstandardized B | Coefficient  Std. Error | R2 change | Significance  (*P*) |
| --- | --- | --- | --- | --- | --- |
|  | (Constant) | 36.42 | 4.78 |  | <0.001 |
|  | BBT | 0.36 | 0.14 | 0.67 | 0.120 |
|  | & Maximal grip force | 11.67 | 8.42 | 0.03 | 0.169 |
| 1 R2=0.79 (0.78) | & Release duration | -0.04 | 0.01 | 0.09 | <0.001 |
|  |  |  |  |  |  |
|  | (Constant) | 36.42 | 4.78 |  | <0.001 |
|  | BBT | 0.36 | 0.14 | 0.67 | 0.012 |
|  | & Release duration | 11.67 | 0.01 | 0.11 | <0.001 |
| 2 R2=0.79 (0.78) | & Maximal grip force | -0.043 | 8.42 | 0.005 | 0.169 |
|  |  |  |  |  |  |
| Controlling for sensory function | | | | | |
|  | (Constant) | 6.94 | 1.99 |  | 0.001 |
|  | BBT | 0.47 | 0.16 | 0.67 | 0.003 |
|  | & Two-point discrimination. | 11.68 | 3.42 | 0.05 | 0.001 |
| 3 R2=0.74 (0.73) | & Maximal grip force | 21.81 | 9.08 | 0.02 | 0.018 |
|  |  |  |  |  |  |
|  | (Constant) | 6.76 | 1.92 |  | 0.001 |
|  | BBT | 0.47 | 0.12 | 0.67 | <0.001 |
|  | & Two-point discrimination. | 9.23 | 3.47 | 0.05 | 0.009 |
| 4 R2=0.75 (0.74) | & Dexterity score | 33.77 | 9.45 | 0.04 | 0.001 |
|  |  |  |  |  |  |
|  | (Constant) | 20.41 | 3.32 |  | <0.001 |
|  | BBT | 0.70 | 0.08 | 0.67 | <0.001 |
|  | & Two-point discrimination. | 9.72 | 3.21 | 0.05 | 0.003 |
| 5 R2=0.78 (0.77) | & Tracking error | -4.72 | 1.03 | 0.06 | <0.001 |
|  |  |  |  |  |  |
|  | (Constant) | 34.33 | 4.29 |  |  |
|  | BBT | 0.40 | 0.09 | 0.67 | <0.001 |
|  | & Two-point discrimination. | 10.96 | 2.83 | 0.05 | <0.001 |
| 6 R2=0.82 (0.81) | & Release duration | -0.04 | 0.01 | 0.10 | <0.001 |
|  |  |  |  |  |  |
| Controlling for wCST-LL | | | | | |
|  | (Constant) | 20.94 | 3.25 |  | <0.001 |
|  | BBT | 0.44 | 0.16 | 0.66 | 0.006 |
|  | & wCST-LL | -1.87 | 0.48 | 0.06 | <0.001 |
| 7 R2=0.74 (0.73) | & Maximal grip force | 20.60 | 9.13 | 0.02 | 0,027 |
|  |  |  |  |  |  |
|  | (Constant) | 19.16 | 3.13 |  | <0.001 |
|  | BBT | 0.39 | 0.12 | 0.66 | 0.002 |
|  | & wCST-LL | -1.74 | 0.46 | 0.06 | <0.001 |
| 8 R2=0.77 (0.76) | & Dexterity score | 35.65 | 9.15 | 0.05 | <0.001 |
|  |  |  |  |  |  |
|  | (Constant) | 28.74 | 3.43 |  | <0.001 |
|  | BBT | 0.72 | 0.08 | 0.66 | <0.001 |
|  | & wCST-LL | -1.14 | 0.52 | 0.06 | 0.033 |
| 9 R2=0.76 (0.75) | & Tracking error | -4.29 | 1.24 | 0.04 | 0.001 |
|  |  |  |  |  |  |
|  | (Constant) | 40.39 | 4.51 |  | <0.001 |
|  | BBT | 0.48 | 0.09 | 0.66 | <0.001 |
|  | & wCST-LL | -1.10 | 0.47 | 0.06 | 0.021 |
| 10 R2=0.79 (0.78) | & Release Duration | -0.04 | 0.01 | 0.07 | <0.001 |
|  |  |  |  |  |  |
| Controlling for hand spasticity | | | | | |
|  | (Constant) | 13.10 | 2.35 |  | <0.001 |
|  | BBT | 0.52 | 0.16 | 0.67 | 0.002 |
|  | & Hand spasticity (NC) | -0.66 | 0.28 | 0.02 | 0.019 |
| 11 R2=0.72 (0.71) | & Maximal grip force | 24.98 | 9.27 | 0.02 | 0.009 |
|  |  |  |  |  |  |
|  | (Constant) | 11.75 | 2.28 |  | <0.001 |
|  | BBT | 0.48 | 0.12 | 0.67 | <0.001 |
|  | & Hand spasticity (NC) | -0.58 | 0.26 | 0.03 | 0.031 |
| 12 R2=0.75 (0.74) | & Dexterity score | 39.07 | 9.15 | 0.06 | <0.001 |
|  |  |  |  |  |  |
|  | (Constant) | 26.59 | 3.18 |  | <0.001 |
|  | BBT | 0.77 | 0.07 | 0.67 | <0.001 |
|  | & Hand spasticity (NC) | 0-537 | 0.26 | 0.02 | 0.038 |
| 13 R2=0.76 (0.76) | & Tracking error | -5.10 | 1.04 | 0.07 | <0.001 |
|  |  |  |  |  |  |
|  | (Constant) | 39.08 | 4.42 |  | <0.001 |
|  | BBT | 0.51 | 0.09 | 0.67 | <0.001 |
|  | & Hand spasticity (NC) | -0.34 | 0.25 | 0.02 | 0.164 |
| 14 R2=0.79 (0.78) | & Release Duration | -0.04 | 0.01 | 0.09 | <0.001 |
|  |  |  |  |  |  |
| Controlling for FMA-UE | | | | | |
|  | (Constant) | 2.36 | 1.87 |  | 0.212 |
|  | FMA-UE | 1.14 | 0.15 | 0.79 | <0.001 |
| 15 R2=0.80 (0.79) | & Maximal grip force | -10.88 | 9.48 | 0.003 | 0.255 |
|  |  |  |  |  |  |
|  | (Constant) | 3.239 | 1.818 |  | 0.078 |
|  | FMA-UE | 0.871 | 0.127 | 0.79 | <0.001 |
| 16 R2=0.80 (0.79) | & Dexterity score | 10.810 | 10.036 | 0.003 | 0.285 |
|  |  |  |  |  |  |
|  | (Constant) | 11.251 | 3.306 |  | 0.001 |
|  | FMA-UE | 0.882 | 0.061 | 0.79 | <0.001 |
| 17 R2=0.81 (0.81) | & Tracking error | -2.873 | 0.987 | 0.02 | 0.005 |
|  |  |  |  |  |  |
|  | (Constant) | 21.864 | 5.610 |  | <0.001 |
|  | FMA-UE | 0.701 | 0.094 | 0.793 | <0.001 |
| 18 R2=0.83 (0.83) | & Release Duration | -0.027 | 0.008 | 0.03 | 0.001 |

**Abbreviations**: BBT – Box and Block Test, wCST-LL – weighted Corticospinal Tract Lesion Load, FMA-UE – Fugl-Meyer Assessment for the Upper Extremity. Each set of models were designed to control if the effect of grip force control variables on BBT outcome remained when adding sensory function, CST lesion load, hand spasticity and FMA-UE, one at a time, to the respective model (see Table 5). E.g. in model 1, BBT at T1 and sensory function (two-point discrimination) at T1 are combined with Maximal grip force, in model 2 with Dexterity-score and so forth. In model 13 to 16, the effect of FMA-UE was tested with BBT excluded to avoid co-variability between these two variables. Unstandardized beta (B) expresses the slope of the regression line, i.e. with each unit change in the independent variable, the dependent variable will change with B. Coefficient Std. Error represents the standard deviation of the coefficient (B) and informs about the precision of the estimate, and R2 indicates the proportion of variance explained by the model (adjusted R2 in parentheses).

Note regarding risk of over-fitting of models: we report the non-adjusted R square since we wanted to report the added contribution of each added variable to the total variance explained and since the R square change is calculated according to non-adjusted R square parameters. The non-adjusted R square can increase with each added term to the model, irrespective of its correlation with the dependent variable while the adjusted R square will decrease if the added variable does not contribute significantly to the model. In order to avoid this problem of over-fitting the models, we only accepted explanatory variables that resulted in a significant R square change (increase in adjusted R square). Hence, in the presented models, the non-adjusted and adjusted R squares were comparable.

**Supplementary Table 5** Associations (correlations) between grip force control measures and measures of structural and functional connectivity.

|  | | Max. grip force | | | | | Dexterity score | | | | | Tracking error | | | | | Release duration | | | |
| --- | --- | --- | --- | --- | --- | --- | --- | --- | --- | --- | --- | --- | --- | --- | --- | --- | --- | --- | --- | --- |
|  | | T1 | T2 | T3 | Δ | T1 | | T2 | T3 | Δ | T1 | | T2 | T3 | Δ | T1 | | T2 | T3 | Δ |
| wCST-LL | T1 | -0.555* | -0.631* | -0.644* | -0.217 | -0.536* | | -0.651* | -0.673* | -0.326* | 0.614* | | 0.650* | 0.614* | -0.069 | 0.642* | | 0.709* | 0.647* | -0.030 |
| Functional Connectivity (FC) | | | | | | | | | | | | | | | | | | | | |
| M1-M1 | T1 | 0.183 | 0.172 | 0.163 | -0.051 | 0.112 | | 0.178 | 0.175 | 0.125 | -0.133 | | -0.105 | -0.174 | -0.066 | -0.020 | | -0.171 | -0.177 | -0.258 |
| M1-SMA | T1 | 0.242 | 0.272 | 0.284 | 0.069 | 0.152 | | 0.214 | 0.193 | 0.086 | -0.258 | | -0.248 | -0.201 | 0.122 | -0.148 | | -0.278 | -0.221 | -0.122 |
| M1-aIPS | T1 | 0.008 | -0.048 | -0.039 | -0.094 | -0.056 | | -0.026 | -0.010 | 0.086 | 0.091 | | 0.094 | -0.026 | -0.217 | 0.197 | | 0.070 | 0.046 | -0.238 |
| M1-vPMC | T1 | 0.068 | 0.087 | 0.055 | -0.032 | 0.097 | | 0.098 | 0.136 | 0.070 | 0.068 | | 0.01 | -0.009 | -0.143 | -0.068 | | -0.017 | -0.093 | -0.044 |
| M1-dPMC | T1 | 0.059 | 0.066 | 0.051 | -0.022 | 0.083 | | 0.065 | 0.116 | 0.061 | 0.068 | | 0.031 | -0.005 | -0.135 | 0.010 | | 0.034 | -0.049 | -0.096 |
| M1- RCZ | T1 | 0.092 | 0.077 | 0.085 | -0.022 | 0.051 | | 0.162 | 0.179 | 0.237 | -0.144 | | -0.134 | -0.068 | 0.151 | -0.098 | | -0.100 | -0.170 | -0.120 |
| Functional Connectivity (FC) | | | | | | | | | | | | | | | | | | | | |
| M1-M1 | T3 | 0.140 | 0.180 | 0.293 | 0.306 | 0.202 | | 0.272 | 0.267 | 0.142 | -0.183 | | -0.120 | -0.147 | 0.076 | -0.116 | | -0.297 | -0.339 | -0.360 |
| M1-SMA | T3 | 0.011 | 0.103 | 0.174 | 0.323 | -0.045 | | 0.111 | 0.124 | 0.303 | -0.393 | | -0.408 | -0.435* | -0.060 | -0.149 | | -0.296 | -0.363 | -0.334 |
| M1-aIPS | T3 | 0.119 | 0.096 | 0.154 | 0.054 | 0.154 | | 0.222 | 0.237 | 0.174 | 0.072 | | 0.123 | 0.092 | 0.036 | -0.076 | | -0.180 | -0.188 | -0.156 |
| M1-vPMC | T3 | 0.190 | 0.204 | 0.197 | 0.002 | 0.254 | | 0.321 | 0.305 | 0.099 | -0.166 | | -0.202 | -0.134 | 0.062 | -0.237 | | -0.288 | -0.253 | -0.034 |
| M1-dPMC | T3 | 0.219 | 0.213 | 0.228 | -0.001 | 0.251 | | 0.244 | 0.233 | -0.024 | -0.016 | | -0.107 | -0.118 | -0.189 | -0.299 | | -0.224 | -0.212 | 0.130 |
| M1-RCZ | T3 | -0.101 | -0.162 | -0.129 | -0.052 | -0.087 | | -0.087 | -0.103 | -0.036 | -0.149 | | -0.171 | -0.069 | 0.158 | 0.033 | | 0.049 | 0.102 | 0.111 |

**Abbreviations**: wCST-LL – weighted Corticospinal Tract Lesion Load, M1 – primary motor cortex (hand area), SMA – supplementary motor area, aIPS – anterior intraparietal sulcus, vPMC – ventral premotor cortex, dPMC – dorsal premotor cortex, RCZ – rostral cingulate zone. Functional connectivity measures (FC) are interhemispheric (M1-M1) and intrahemispheric of the lesioned (contralateral) hemisphere. Numbers are Pearson correlation coefficients. * indicate statistically significant coefficients when corrected for multiple comparisons.
